# A Novel Bacterium-Like Particle Vaccine Displaying the MERS-CoV Receptor-Binding Domain Induces Specific Mucosal and Systemic Immune Responses in Mice

**DOI:** 10.3390/v11090799

**Published:** 2019-08-29

**Authors:** Entao Li, Hang Chi, Pei Huang, Feihu Yan, Ying Zhang, Chuanyu Liu, Zhenshan Wang, Guohua Li, Shengnan Zhang, Ruo Mo, Hongli Jin, Hualei Wang, Na Feng, Jianzhong Wang, Yuhai Bi, Tiecheng Wang, Weiyang Sun, Yuwei Gao, Yongkun Zhao, Songtao Yang, Xianzhu Xia

**Affiliations:** 1 College of Veterinary Medicine, South China Agricultural University, Guangzhou 510642, China; 2 Changchun Veterinary Research Institute, Chinese Academy of Agricultural Sciences, Changchun 130000, China; 3 Key Laboratory of Jilin Province for Zoonosis Prevention and Control, Changchun 130000, China; 4 Animal Science and Technology College, Jilin Agricultural University, Changchun 130118, China; 5 College of Wildlife and Protected Area, Northeast Forestry University, Harbin 150040, China; 6 College of Animal Science and Technology, Shihezi University, Shihezi 832003, China; 7 College of Veterinary Medicine, Jilin University, Changchun 130062, China; 8 CAS Key Laboratory of Pathogenic Microbiology and Immunology, Institute of Microbiology, Chinese Academy of Sciences, Beijing 100101, China

**Keywords:** MERS-CoV, subunit vaccine, bacterium-like particles, intranasal administration, mucosal immune

## Abstract

Middle East respiratory syndrome coronavirus (MERS-CoV), a new coronavirus that has been causing severe and fatal acute respiratory illnesses in humans since its outbreak in 2012, has raised public fear worldwide. The development of prophylactics and therapeutics is urgently needed to prevent and control MERS-CoV infections. In this study, a bacterium (*Lactococcus lactis*)-like particle (BLP) vaccine displaying the MERS-CoV receptor-binding domain (RBD) was developed, and gram-positive enhancer matrix (GEM) particles were used as substrates to externally bind to the MERS-CoV RBD through a protein anchor (PA). The designs included different numbers of lysin motif (LysM) repeats in the PAs linked by linkers (RBD-linker-PA2 (RLP_2_), RBD-linker-PA3 (RLP_3_) and RBD-PA3 (RP_3_)), and three LysM repeats and a linker in the fusion proteins increased the binding activity to the RBD. The specific immune responses were tested by intranasally immunizing mice with RLP_3_-GEM with or without the adjuvant GEL01. The results showed that GEL01-adjuvanted RLP_3_-GEM increased the systemic humoral, cellular and local mucosal immune responses in the mouse model, especially in the intestinal tract. The above results indicate that the MERS-CoV BLP product has the potential to be developed into a promising mucosal candidate vaccine to protect against MERS-CoV infections.

## 1. Introduction

Middle East respiratory syndrome coronavirus (MERS-CoV), a beta coronavirus, causes severe and lethal acute respiratory disease in humans and is remarkably different from other human coronaviruses, including HCoV-229E, HCoVNL63, HCoV-HKU1, and HCoV-OC43, which are known to cause mild respiratory infections [1,2]. Until May 2019, the World Health Organization (WHO) had received 2428 laboratory-confirmed cases from 27 countries after MERS-CoV was identified in 2012; these cases included 838 deaths related to MERS-CoV infections and had a case fatality rate of 35% [3]. In May 2015 the MERS outbreak in South Korea, which was the largest outbreak outside the Middle East and caused 36 deaths in 186 cases from a single infected person [4,5]; this outbreak raised the concerns of a potential global MERS-CoV pandemic. However, there are currently no licensed therapeutic drugs or prophylactic vaccines available to protect against MERS-CoV. Vaccination is the best way to control and prevent infectious diseases [6]. Since MERS-CoV is a respiratory pathogen, any MERS-CoV vaccination that induces antigen-specific secretory IgA (sIgA) antibodies at the mucosal surfaces probably has the effects of preventing MERS-CoV replication at the site of virus infection [7]. The mode of MERS-CoV transmission is not clearly understood, but one study has proven that the human intestinal tract may be a new transmission route for MERS-CoV [8], indicating that antigen-specific sIgA antibodies in the gastrointestinal mucosa are a considerable indicator. Therefore, there is an urgent need to develop an effective mucosal immune vaccine against MERS-CoV, especially those inducing gastrointestinal mucosal immunity.

Up to date, researchers have developed a number of promising approaches for MERS-CoV vaccine candidates, including DNA vaccines [9,10,11], recombinant viral vectors [12,13,14,15,16,17,18], and protein-based platforms [6,19]. However, no vaccines have entered clinical trials except for a DNA vaccine that completed phase I clinical trials [20]. Each of these different MERS-CoV vaccine approaches has its own advantages and disadvantages, such as immunogenicity, ease of mass production, and preexisting immunity in addition to having potential adverse effects. These factors must be taken into account and balanced to create a successful vaccine for use in humans and/or animals. Subunit vaccines, consisting of the major pathogen antigenic fragments, possess many advantages, such as minimal side effects, high safety profiles, and a high yield [21,22]. Until now, some studies have shown that the receptor-binding domain (RBD) of the MERS-CoV spike (S) protein was a major antigenic determinant for the induction of neutralizing antibodies and as an essential target for a MERS subunit vaccine [7,22,23,24,25,26]. Meanwhile, a previous study has identified that the most stable and neutralizing viral RBD fragment is S377-588 from the five following known versions: S350-588, S358-588, S367-588, S367-606 and S377-588 [27]. Therefore, S377-588 of the MERS-CoV S protein is potentially useful for designing an effective vaccine.

Mucosal vaccination of subunit vaccines requires antigen delivery vectors and adjuvants for optimal immune responses [28]. Displaying heterologous pathogen proteins on the surface of bacteria is a rational way to enhance the immunogenicity of a vaccine [29]. A novel surface display system contains nonliving and genetically unmodified gram-positive enhancer matrix (GEM) particles and a protein anchor (PA) from the *Lactococcus lactis* peptidoglycan hydrolase AcmA, which is the GEM-PA surface display system. The GEM-PA surface display system is a flexible, effective, inexpensive, and easy-to-handle alternative for heterologous proteins and peptides on GEM particles [30]; this system has been applied to parasite oral vaccines [31], Streptococcus pneumoniae mucosal vaccines [32], and porcine circovirus type 2 vaccines [33], among others.

The PA of the system surface display is the lysin motif (LysM) derived from the C-terminal peptidoglycan-binding domain of AcmA, an autolysin from *Lactococcus lactis*, and binds to GEM particles in a noncovalent manner [34,35]. The number of LysMs in proteins affects the efficiency of foreign proteins binding to the GEM [34]. Some studies showed that three LysM domains in fusion proteins have optimal peptidoglycan-binding activities and biological functions [36,37]. A previous study shown that two LysM domains in fusion proteins have a much stronger binding activity than the others [30]. Therefore, further study on the number of LysMs in proteins is necessary. LysM domains are often parted from the other domains and from each other through linker sequences, conveying LysM domain mobility and flexibility to the fused target protein and allowing the proteins to attain an optimal orientation in binding with the cell wall [35]. However, the linker peptides between the PA and antigen proteins have yet to be explored for their influence on the binding affinity and immunogenicity of the fusion protein.

Considering that the number of LysMs and linker sequences in the fusion proteins may influence PA binding efficacy to GEM particles and the immunogenicity of the antigen proteins, we expressed RLP_2_, RLP_3_, and RP_3_ based on the recombinant baculovirus expression system. Then, the fusion proteins were tested for their ability to bind to GEM particles, and the immunogenicity of the antigen proteins was tested. The mucosal immunity and systemic immune responses caused by the MERS-CoV BLP candidate vaccine were also detected through intranasal immunization in a mouse model with or without GEL01 adjuvant.

## 2. Materials and Methods

### 2.1. Construction and Expression of Recombinant Baculovirus

The construction of rBV-RLP_2_, rBV-RLP_3_, and rBV-RP_3_ was as follows: MERS-CoV RBD genes encoding S glycoprotein residues 377-588 were amplified by PCR using codon-optimized S (GenBank: KF600645) as a template with oligonucleotide primers RBD-F and linker-RBD-R (Table 1). PA2 genes encoding two LysMs were amplified by PCR using codon-optimized pUC57-PA3 (*L. lactis* MG1363) as a template with the oligonucleotide primers linker-PA2-F and PA3-R. The PCR fragment of RLP_2_ was amplified by a second PCR using the last products of the RBD and PA2 genes as templates with the oligonucleotide primers RBD-F and PA3-R. The resulting construct was digested with XbaI and KpnI and then inserted into the pFastBac1-HBM plasmid (Invitrogen, Carlsbad, CA, USA) to generate the recombinant plasmid pFastBac1-RLP_2_. To construct the recombinant genes RLP_3_ and RP_3_, the genes were amplified by PCR and inserted into the pFastBac1-HBM plasmid with XbaI and KpnI digestion, generating the recombinant plasmids pFastBac1-RLP3 and pFastBac1-RP3, respectively. PFastBac1-RLP_2_, pFastBac1-RLP_3_ and pFastBac1-RP_3_ were then separately transformed into *E. coli* DH10Bac cells to generate recombinant bacmids (rBacmid-RLP_2_, rBacmid-RLP_3_ and rBacmid-RP_3_). Then, the recombinant bacmids were transfected into *Spodoptera frugiperda* 9 (Sf9, Gibco, Grand Island, NY, USA) insect cells using liposome 3000 according to the Bac-to-Bac expression system manual (Invitrogen, USA) and cultured in 6-well plates at 2 × 10^6^ cells/mL to generate the recombinant baculoviruses, rBV-RLP_2_, rBV-RLP_3_, or rBV-RP_3_. Supernatants containing recombinant baculovirus were harvested at 4 days after transfection as viral stocks.

### 2.2. Immunofluorescence Assay (IFA) and Western Blotting Analyses of Recombinant Baculoviruses

An IFA was performed to confirm the expression of RLP_2_, RLP_3_ and RP_3_ as previously described [4]. Briefly, Sf9 cells cultured in 96-well plates at 2 × 10^6^ cells/mL were infected with the recombinant baculovirus. After 48 h of infection, the cultured plates were fixed with 80% cold acetone overnight at -20°C, washed three times with PBS-0.05% Tween 20 (PBST), and then incubated with a rabbit anti-MERS-S polyclonal antibody (1:500, Sino Biological Inc, Beijing, China) containing 1% bovine serum albumin (BSA, Sigma-Aldrich, USA) at 37°C for 1 h. After three washes with PBST, an FITC-labeled goat against rabbit IgG antibody (1:300, BioWorld, Inc, St. Louis, MN, USA) was added with Evans blue (Sigma-Aldrich, St. Louis, MN, USA) for 1 h at 37 °C. After washing, the cells were observed with a fluorescence microscope.

For Western blotting analysis, the culture of Sf9 cells infected with each recombinant baculovirus (rBV-RLP_2_, rBV-RLP_3_, rBV- RP_3_, and rBV) was centrifuged at 6000× *g* at 4 °C for 15 min, and then the culture supernatants were obtained as supernatant fractions. Cell pellets were washed three times with 10 mM PBS (pH 7.2–7.4) and then resuspended in PBS. Samples of supernatant fractions and cell pellets were transferred onto a polyvinylidene fluoride (PVDF) membrane (Merck Millipore, Billerica, MA, USA) after SDS-PAGE under denaturing conditions for Western blotting with a rabbit anti-MERS-CoV-S polyclonal antibody.

### 2.3. Binding of the Fusion Proteins to Gem Particles

GEM particles were prepared as described in detail elsewhere [28]. In brief, cells from the *L. lactis* strain MG1363 were harvested and washed with PBS and then boiled in 10% trichloroacetic acid for 30 min, generating the so-called GEM particles. With a Bürker–Turk counting chamber, the number of GEM particles per milliliter was counted. One unit (U) was defined as 2.5 × 10^9^ GEM-+ particles. The standard procedure was followed, and one unit of GEM particles was added into 10 mL of each recombinant baculovirus culture supernatant; the culture was slowly mixed on a rotary shaker at room temperature for 60 min, generating RLP2-GEM, RLP3-GEM and RP3-GEM. Then, the binding GEM particles were concentrated at 6000× *g* for 10 min at 4 °C, washed and resuspended in sterile PBS, and stored at a concentration of 200 µL/U at −20°C until further use.

### 2.4. SDS-PAGE, Western Blotting and IFA Analysis of the Binding GEM Particles

For the SDS-PAGE and Western blotting analyses of the binding GEM particles, the complexes were resuspended in 5× SDS-PAGE sample buffer (Beyotime Biotechnology, Shanghai, China), separated by 12% SDS-PAGE and then transferred by electroblotting onto PVDF transfer membranes under denaturing conditions for Western blotting with a rabbit anti-MERS-CoV-S polyclonal antibody.

For the IFA, 100 µL binding GEM particles was concentrated at 6000× *g* for 10 min at 4 °C and resuspended in 3% BSA for blocking for 30 min at 37 °C.Then, the binding GEM particles were incubated with a 1:200 dilution of the rabbit anti-MERS-CoV-S polyclonal antibody in PBS with 1% BSA for 60 min at 37 °C. After three washes, the complexes were incubated with an FITC-labeled goat anti-rabbit IgG antibody for 1 h at 37 °C and then viewed and photographed using a Zeiss microscope with incident UV illumination and the Zeiss Axiovision digital imaging system (Zeiss, Oberkochen, Germany).

For the maximum binding capacity of each fusion protein binding the GEM particles, 0.5 U GEM particles were incubated for 60 min at room temperature with 0, 2, 4, 6, 8 and 10 mL of each recombinant baculovirus culture supernatant. Then, SDS-PAGE was used to analyze each fusion protein binding the GEM particles with Gel Image System analysis software, version 4.2 (Tanon, Shanghai, China). Meanwhile, the amount of each fusion protein binding the GEM particles was determined densitometrically by analysis of scans of Coomassie brilliant blue-stained SDS–12% polyacrylamide (PAA) gels with the Quantity One image analysis software, version 4.6.7. A calibration curve was generated using BSA protein standards on the same PAA gel.

### 2.5. Immunizations and Samples Collection

A total of two batches of BALB/c mice (6 weeks old, female) were procured from the Changchun Yisi Laboratory Animal Technology Co., Ltd. (Changchun, China) and were immunized. In Batch I, twenty mice were randomly distributed into four groups (*n* = 5 per group) and vaccinated intramuscularly (IM). Mice in group 1 were vaccinated with PBS as a control; mice in group 2 were vaccinated with GEM adjuvanted with a complex of ISA201VG (Seppic, Paris, France) and PolyI:C (Sigma, St. Louis, MN, USA) as a control; mice in group 3 were vaccinated with 5 μg RP_3_-GEM antigen proteins adjuvanted with the same in group 2; mice in group 4 were vaccinated with 5 μg RLP_3_-GEM antigen proteins adjuvanted with the same in group 2. All the groups were boosted twice with the same 3-week intervals. Blood samples were collected at two, five and eight weeks postimmunization (wpi).

In Batch II, thirty mice were randomized into three groups and vaccinated intranasally (IN). Mice in group 1 were vaccinated with PBS as a control; mice in group 2 were vaccinated with 5 μg RLP_3_-GEM antigen proteins; mice in group 3 were vaccinated with 5 μg RLP_3_-GEM antigen proteins mixed with 10 μL GEL01 adjuvant (Seppic, Paris, France). All the groups were boosted twice with the same 3-week intervals. Blood samples were collected at two, five and eight weeks wpi. Serum samples were inactivated at 56℃ for 30 min prior to analysis. The lung lavage samples were collected with 1 mL cold PBS, and the gut-wash samples were collected with 2 mL cold PBS on ice at eight wpi. The sera, lung lavage and gut-wash sample supernatants were collected after centrifugation at 6000× *g* for 15 min at 4 °C and were stored at −80 °C for further use.

### 2.6. Pseudotyped Virus Neutralization Assay

A pseudotyped virus neutralization assay was performed as described previously [38]. In brief, 100× TCID_50_ of MERS-pseudotyped virus was mixed with an equal volume of serially diluted mouse sera, and the mixtures were incubated at 37 °C for 30 min and then incubated with Huh 7 cells for 4 h. Each sample was assayed in quadruplicate. The incomplete medium was replaced with complete DMEM (10% fetal bovine serum+1% penicillin-streptomycin), and then the samples were incubated at 37 ℃ for 48 h. The luciferase activity of the samples was measured with an Infinite M200 Microplate Spectrophotometer (Tecan, Männedorf, Switzerland).

### 2.7. Enzyme-Linked Immunosorbent Assay (ELISA) Measurement of RBD-Specific Antibodies

The lung lavage, gut-wash and sera samples from the animals were collected at eight wpi and tested for RBD-specific IgA, IgG, IgG1 and IgG2a antibodies by an ELISA. Briefly, purified RBD antigen produced in *E.coli* (1 μg/mL) in 100 μL carbonate buffer was used to coat 96-well microtiter plates (Corning-Costar, Corning, NY, USA) overnight at 4 ℃. Following three washes with PBST and blocking with PBST containing 3% BSA for 2 h at 37 °C, the plates were incubated with 2-fold serial dilutions of samples in PBS containing 0.5% (*w/v*) BSA at 37 °C for 1 h. After three wash cycles with PBST, the plates were incubated with the following HRP-labeled goat antibodies: anti-mouse IgA (1:2000, SouthernBiotech, Birmingham, AL, USA), anti-mouse IgG (1:2000, BioWorld, St. Louis, MN, USA), anti-mouse IgG1 (1:2000, Southern Biotech, USA), and anti-mouse IgG2a (1:2,000, SouthernBiotech, Birmingham, AL, USA) at 37°C for 1 h. Subsequently, the plates were washed three times and 100 µL tetramethylbenzidine substrate was added per well; the color development was stopped by adding 50 µL/well H_2_SO_4_. Optical density values were measured at 450 nm using an ELISA plate reader (Bio-Rad, Hercules, CA, USA).

### 2.8. ELISpot IFN-γ and IL-4 Cytokine Assays

ELISpot IFN-γ and IL-4 cytokine assays were performed as described previously [9]. Splenocytes were harvested in complete RPMI 1640 medium at 7 days after the third immunization and plated in a 96-well ELISpot plate (MABTECH, Nacka, Sweden). Purified RBD antigen produced in *E.coli* was or was not added to each well at a final concentration of 10 μg/mL to stimulate cytokine production. After incubation for 40 h, IFN-γ and IL-4 were detected using mouse enzyme-linked immunospot (ELISpot) kits according to the manufacturer’s instructions. Spot-forming cells (SFCs) were counted with an ELISpot reader (Multispotreader Spectrum, AID, Strasberg, Germany).

### 2.9. Splenocyte Proliferation Assay

The splenocyte proliferation assay was performed as described [39]. In brief, splenocytes were stimulated with or without purified RBD antigen (10 µg/mL) produced in *E.coli* at a concentration of 2.5 × 10^6^ cells/mL in triplicate (100 μL/well) in a 96-well plate. After incubation at 37 ℃ and 5% CO_2_ for 44 h, 10 µL of CCK-8 solution (KeyGEN Biotech, Nanjing, China) was added to each well. After incubation for an additional 4 h, the plates were measured at 450 nm using an Infinite M200 Microplate Spectrophotometer. The formula for the proliferation index (PI) was expressed as follows:

PI = (OD for stimulated cultures − OD for non-stimulated cultures)/(OD for non-stimulated cultures − OD for control cultures).

### 2.10. Evaluation of B Cell and T Cell Activation by Flow Cytometry

Frequencies of activated B cells and T cells in splenocytes were evaluated by flow cytometry. Splenocytes were cultured in complete RPMI 1640 and stimulated with RBD antigen (10 μg/mL) produced in *E.coli* for 60 h. Cells were then stained with the following anti-mouse antibodies: APC-anti-CD19, FITC-anti-CD4, PE-anti-CD8, PE/Cy7-anti-CD69 (BD Biosciences, San Jose, CA, USA). After washing, the labeled cell samples were examined by a FACSAriaTM Cell Sorter (BD Biosciences, San Jose, CA, USA).

### 2.11. ELISA Measurement of Cytokine Levels in Splenocyte Culture Supernatants

Splenocytes were harvested 7 days after the third immunization and stimulated with RBD antigen (10 μg/mL) produced in *E.coli* for 72 h at 37 °C and 5% CO_2_. The supernatant was collected by centrifugation (600× *g*, 10 min). T helper 1 (Th1) cytokines (tumor necrosis factor [TNF]-α, interferon [IFN]-γ, and interleukin [IL]-2) and Th2 cytokines (IL-4, IL-6, and IL-10) in the supernatants were detected using mouse ELISA cytokine kits (MABTECH, Nacka, Sweden) according to the manufacturer’s instructions.

The purified RBD antigen has been tested to produce similar background responses in PBS or GEM immunized animals.

### 2.12. Data Analysis

The results are expressed as the means ± SD. Figures were generated using GraphPad Prism 8.0 software (GraphPad Software Inc.). Significance differences between the groups were analyzed using one-way ANOVA and were deemed significant at P values of 0.05 or less.

### 2.13. Laboratory Facility and Ethics Statement

The treatment of all mice was in accordance with the welfare and ethical guidance of Chinese laboratory animals (GB 14925-2001). The agreement was approved by the Animal Welfare and Ethics Committee of the Institute of Veterinary Medicine of the Military Academy of Sciences (Laboratory Animal Care and Use Committee Authorization, permit number JSY-DW-2018-02).

## 3. Results

### 3.1. Expression of Fusion Proteins

**Figure 1:**
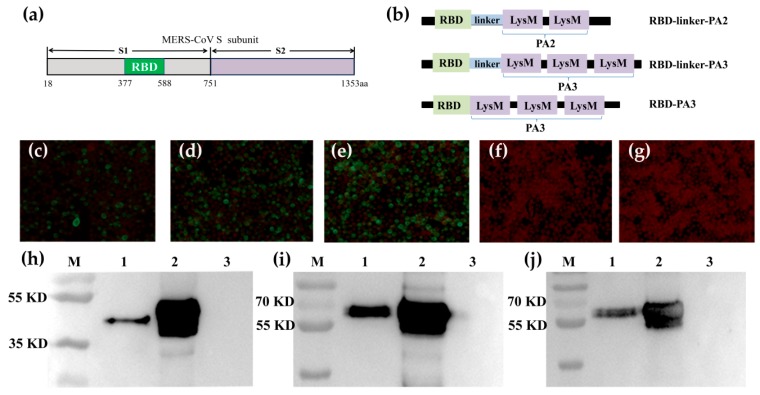
Construction and detection of the fusion proteins expression in baculovirus-infected Sf9 insect cells. (**a**) Schematic illustration of the MERS-CoV-S subunit; (**b**) Schematic illustration of RBD-linker-PA2, RBD-linker-PA3, and RBD-PA3 fusion proteins; (**c**–**g**) IFA detection of the RLP_2_, RLP_3_, and RP_3_ expression in baculovirus-infected Sf9 insect cells (Magnification of microscopy images, ×200). Cells were infected with rBV-RLP_2_, rBV-RLP_3_, rBV-RP_3_ in (**c**–**e**); cells infected with rBV (**f**) and uninfected cells (**g**) were the mock cells. After 48 h, cells were detected with a rabbit anti-MERS-S polyclonal antibody. (**h**–**j**): Western blot analysis of the rBV-RLP_2_ (**h**), rBV-RLP_3_ (**i**), rBV-RP_3_ (**j**) protein expression in Sf9-infected cells. Expression was detected with a rabbit anti-MERS-S polyclonal antibody. M: molecular weight marker, 1: culture supernatant, 2: cell sedimentation, 3: rBV infected cells.

The strategy for designing RLP_2_, RLP_3_, and RP_3_ fusion proteins is shown in Figure 1a, b, in which RBD was fused to PA2 and PA3 with or without a linker. The IFA results showed that compared to the control cells, the Sf9 cells expressing RLP_2_, RLP_3_, and RP_3_ proteins emitted strong green fluorescence signals with an anti-MERS-S polyclonal antibody for RBD, suggesting that the expressed fusion proteins have good antigenicity (Figure 1c–g). Furthermore, Western blotting analysis showed that the RLP_2_, RLP_3_ and RP_3_ recombinant proteins were successfully expressed as soluble proteins and secreted into the supernatants (Figure 1h–j).

### 3.2. Location of Fusion Proteins on GEM Particles

**Figure 2:**
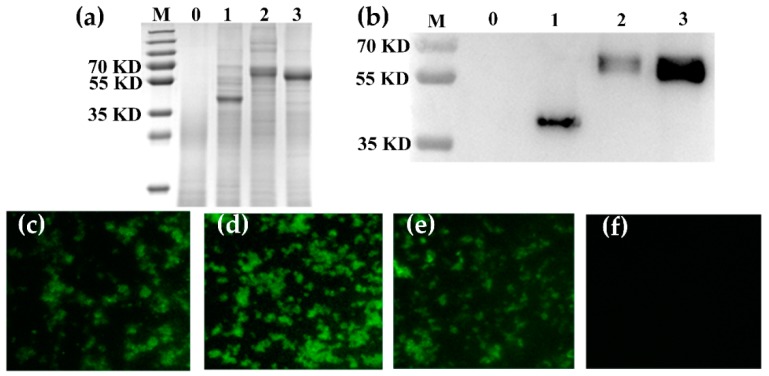
Detection of the fusion proteins displaying the GEM particles. (**a**) SDS-PAGE analysis of the displaying of RLP_2_, RLP_3_, and RP_3_ proteins on GEM particles. Lane 1: GEM particles displaying RLP_2_ from the culture supernatant of rBV-RLP_2_-infected Sf9 cells; Lane 2: GEM particles displaying RLP_3_ from the culture supernatant of rBV-RLP_3_-infected Sf9 cells; Lane 3: GEM particles displaying RP_3_ from the culture supernatant of rBV-RP_3_-infected Sf9 cells; Lane 0: GEM particles; M: molecular weight marker. (**b**) Western blot analysis of the proteins displayed on GEM particles. Lane 1, Lane 2, Lane 3, and Lane 0 are the same as in Figure 2a. (**c**–**f**) Representative fluorescence microscopy images showing that fusion proteins were loaded on GEM particles (Magnification of microscopy images, 1000 ×). GEM particles were bound with RLP_2_ (**c**), RLP_3_ (**d**), and RP_3_ (**e**) proteins and were the mock control (**f**); proteins were detected with a rabbit anti-MERS-S polyclonal antibody.

The surface location of RLP_2_, RLP_3_ and RP_3_ fusion proteins on GEM particles was analyzed by SDS-PAGE (Figure 2a), Western blotting (Figure 2b) and IFA with an anti-MERS-S polyclonal antibody (Figure 2c–f). SDS-PAGE and Western blotting observations showed that the RLP_2_, RLP_3_ and RP_3_ fusion proteins were bound to GEM particles. Meanwhile, immunofluorescence microscopy observations showed that compared to the GEM particles (negative control), the combination of the GEM particles and fusion proteins emitted a strong green fluorescence. Therefore, the above results indicated that the RLP_2_, RLP_3_ and RP_3_ fusion proteins were anchored to GEM particles.

### 3.3. Binding Activity of Fusion Proteins on GEM Particles

**Figure 3:**
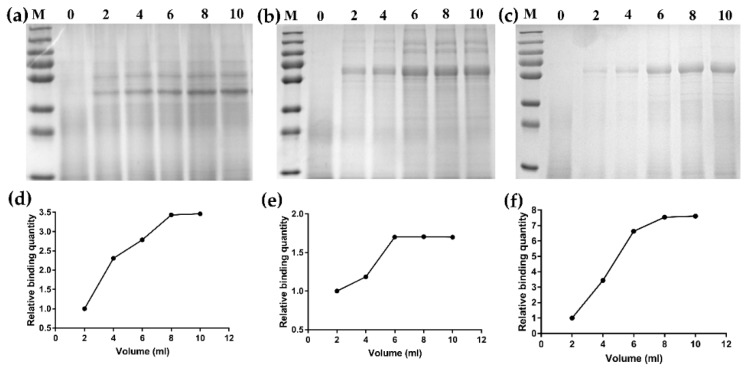
Detection of binding activity of the fusion proteins on GEM particles by SDS-PAGE. (**a**–**c**) The maximum binding capacity of each fusion proteins binding to the GEM particles when 0.5 U GEM particles was combined with 0, 2, 4, 6, 8 and 10 mL of each recombinant baculovirus culture supernatant. (**a**) RLP_2_-GEM; (**b**) RLP_3_-GEM; (**c**) RP_3_-GEM. (**d**–**f**) The relative binding quantity of binding fusion proteins on the GEM particles was detected densitometrically by analysis of scans of PAA gels with Gel Image System analysis software (Tanon, China). M: molecular weight marker; 0: GEM particles.

To investigate whether the differences in binding activity on GEM particles were caused by differences in LysM repeats and the extension of anchor proteins plus a linker to the RBD, direct binding capacity studies on GEM particles was performed by SDS-PAGE (Figure 3). To analyze the maximum binding capacity of each fusion protein binding the GEM particles, 0.5 U GEM particles were combined with 0, 2, 4, 6, 8 and 10 mL of each recombinant baculovirus culture supernatant. According to the relative binding quantity on the Coomassie brilliant blue-stained polyacrylamide gels, with the increase in binding culture supernatant volume of 0.5 U GEM particles, the relative quantity of binding fusion proteins increased, and the relative quantity of all fusion proteins in 8 mL and 10 mL volumes was similar; this result suggests that the GEM particles binding the fusion proteins have been saturated. Furthermore, we estimated that 1U GEM particles can bind 133.2 μg of the RLP_2_ fusion protein, 217.76 μg of the RLP_3_ fusion protein and 191.86 μg of the RP_3_ fusion protein with the Quantity One image analysis software. The above results indicated that the binding activities of RLP_3_ and RP_3_ were better than those of RLP_2_; thus, RLP_3_ and RP_3_ were selected for further experiments in mice.

### 3.4. Virus Neutralizing Antibodies and ELISA Measurement of Subtype Antibodies in Serum

**Figure 4:**
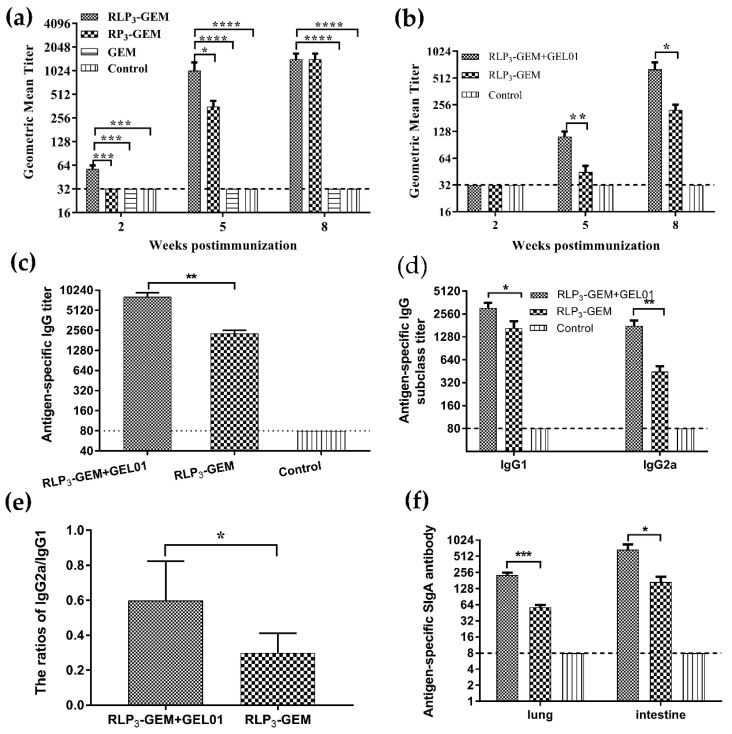
Detection of antibody levels in the serum and mucosa from the immunized mice. Serum samples were collected at weeks two, five, and eight. Neutralizing antibody titers in the serum at weeks two, five, and eight were performed by a MERS-CoV pseudotyped virus neutralization assay. Antigen-specific IgG, IgG1, IgG2a and IgA antibody levels in the serum or in mucosa at week 8 were assessed by indirect ELISA with the purified RBD protein, displaying as the end-point dilution titers. The horizontal dotted line in the figure indicates the limit of determination (LOD). *n* = 5 mice/group/time point. Data are shown as the means ± SD and were analyzed by one-way ANOVA (* *p* < 0.05; ** *p* < 0.01; *** *p* < 0.001; **** *p* < 0.0001). (**a**) The neutralization titers of serum samples from mice immunized intramuscularly at the indicated times with RLP_3_-GEM and RP_3_-GEM. (**b**) The neutralization titers of serum samples from mice immunized intranasally with RLP3-GEM and RLP_3_-GEM+GEL01 at the indicated times. (**c**–**e**) RBD-specific IgG (**c**), IgG1 (**d**) and IgG2a (**d**) titers in the serum from mice immunized intranasally at week 8. Ratios of IgG2a/IgG1 (**e**) were calculated. (**f**) The local immune responses from mice intranasally immunized with RLP_3_-GEM and RLP_3_-GEM+GEL01. Antigen-specific mucosal IgA titers in lung lavage fluid and intestine washes of immunized mice at week 8.

Antibody responses in the serum to MERS-CoV were measured by a pseudotyped virus neutralization assay and shown as end-point dilution titers at two, five, and eight wpi. To enhance the immune response of the RLP_3_-GEM and RP_3_-GEM in mice, we applied a complex of ISA201VG and PolyI:C as adjuvants that we screened out in mice through intramuscular administration in our laboratory. The results showed that the antibody levels of the sera from the RLP_3_-GEM- and RP_3_-GEM-immunized mice gradually increased to plateau at eight weeks, and there were significant differences in the neutralizing activities between the two groups at two and five weeks (Figure 4a). These results suggested that the immunogenicity of RLP_3_-GEM was stronger than that of RP_3_-GEM, and thus, RLP_3_-GEM was selected for further intranasally vaccinated experiments in mice.

To analyze whether intranasally RLP_3_-GEM-immunized mice could produce systemic humoral immune responses, specific serum antibody levels were determined by a pseudotyped virus neutralization assay and ELISA. GEL01 is a commercial adjuvant based on a polymer technology, already applied in the adjuvant for veterinary vaccine field, and can be used in parenteral and mucosal immunity. To decide whether the GEL01 could enhance the immune response of the RLP_3_-GEM in mice or not, we intranasally immunized mice with it. Pseudotyped virus neutralization assay data showed that RLP_3_-GEM and RLP_3_-GEM plus GEL01 induced strong neutralization against an MERS-CoV infection, and the latter displayed a much higher neutralizing titer than the former (Figure 4b). A similar phenomenon was also found for the RBD-specific IgG (Figure 4c), IgG1 (Figure 4d), and IgG2a (Figure 4d) antibodies in the sera at eight wpi caused by RLP_3_-GEM and RLP_3_-GEM plus GEL01. Meanwhile, the ratios of IgG2a/IgG1 of RLP3-GEM plus GEL01 were much higher than those of RLP_3_-GEM (Figure 4e), suggesting that GEL01 could induce a Th1-polarized immune response. Collectively, these data showed that RLP_3_-GEM could induce systemic immune responses and that the adjuvant GEL01 could significantly enhance antibody responses.

### 3.5. Mucosal IgA Levels in Lungs and Intestines

To verify whether RLP_3_-GEM could produce local immune responses in intranasally immunized mice, specific IgA levels in lung lavage fluid and intestinal washes were determined by ELISA at eight wpi. The data showed that mucosal IgA levels were present among some of the tissues and organs in the RLP_3_-GEM and RLP_3_-GEM plus GEL01 groups, and the IgA levels were more obvious in the RLP_3_-GEM plus GEL01 group (Figure 4f). These results showed that mucosal immune responses were induced by the RLP_3_-GEM candidate vaccine, and the effect was significantly increased by the adjuvant GEL01.

### 3.6. Splenocyte Proliferation by ex vivo Restimulation

**Figure 5:**
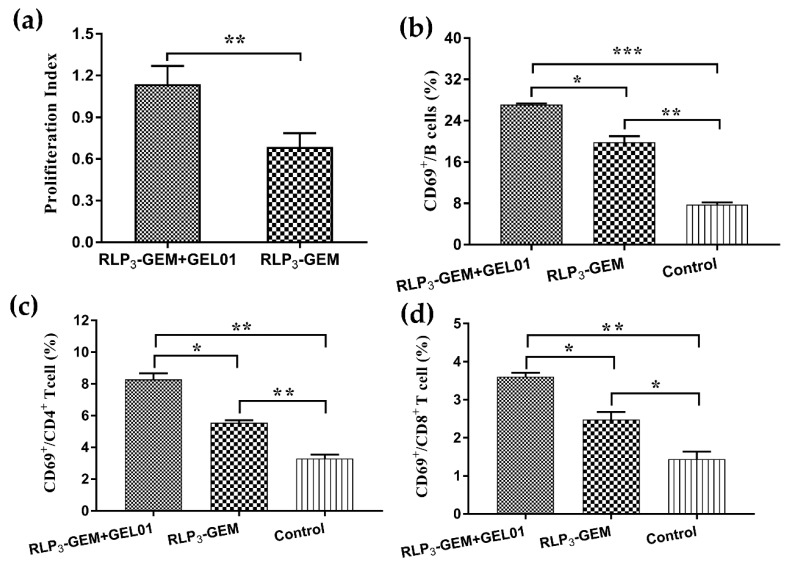
Index of lymphocytes and activated (CD69^+^) B cells, CD4^+^ and CD8^+^ T cells harvested from the spleen. At 7 days after the last immunization, lymphocytes from mice immunized intranasally were harvested and re-stimulated with RBD (10 μg/mL) in vitro. The proliferative index of the spleen was detected using a CCK-8 assay. The frequency of CD69^+^CD19^+^ B cells, CD69^+^CD4^+^ T cells and CD69^+^CD8^+^ T cells was estimated by flow cytometry. Data in (**a**–**d**) are expressed as the mean ± SD for each group. * *p* < 0.05; ** *p* < 0.01; *** *p* < 0.001.

To analyze the effects of GEL01 on splenocyte proliferative responses, an ex vivo splenocyte proliferation assay was performed. Under the stimulation of RBD proteins, splenocytes harvested from mice intranasally immunized with RLP_3_-GEM plus GEL01 proliferated more efficiently than those from mice immunized with RLP_3_-GEM without GEL01 (Figure 5a). Therefore, these data suggested that GEL01 could evoke potent antigen-specific immune responses.

### 3.7. Splenocyte Activation Assays

CD69 is an activation marker of effector immune cells [40]. Therefore, we detected the frequencies of CD69^+^ in splenocytes collected from immunized mice with flow cytometry to analyze the activation of B cells, CD4^+^ T cells and CD8^+^ T cells. The expression of CD69 on B cells (Figure 5b), CD4^+^ T cells (Figure 5c) and CD8^+^ T cells (Figure 5d) from mice immunized with RLP_3_-GEM with GEL01 significantly increased compared with the expression of cells from mice immunized with RLP_3_-GEM. These results suggested that RLP_3_-GEM with GEL01 induced a more potent immune activation than did RLP_3_-GEM, which is very important to initiate the whole immune response.

### 3.8. Cytokine Secretion by Restimulated Splenocytes

**Figure 6:**
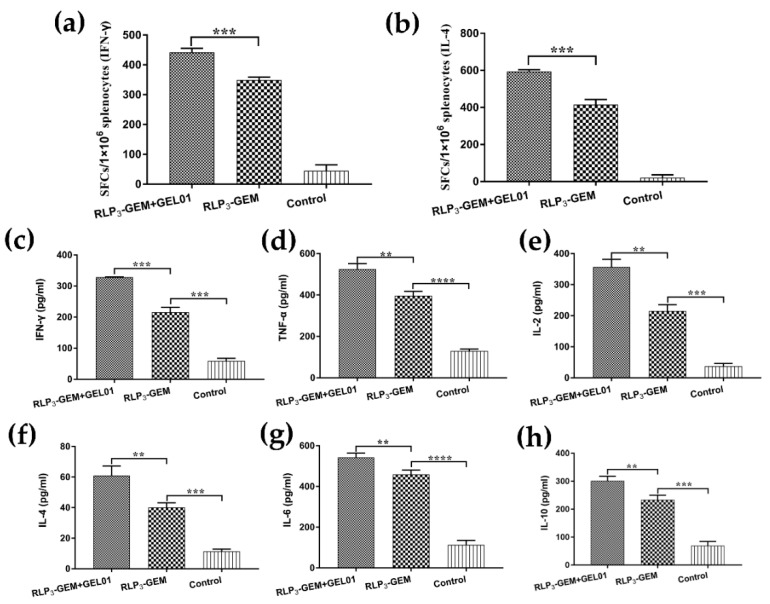
Detection of cytokine secretion levels in splenocytes. Splenocytes were harvested from mice immunized intranasally at 7 days after the last immunization and restimulated with RBD (10 μg/mL) in culture ex vivo. Splenocytes secreting IFN-r (**a**) and IL-4 (**b**) were quantified using an ELISpot assay. Furthermore, the concentrations of IFN-r (**c**), TNF-a (**d**), IL-2 (**e**), IL-4 (**f**), IL-6 (**g**), and IL-10 (**h**) in the supernatant were measured with commercial ELISA kits. Data are expressed as the mean ± SD. * *p* < 0.05; ** *p* < 0.01; *** *p* < 0.001.

Since we analyzed whether RLP_3_-GEM with GEL01 displayed better efficacy than RLP_3_-GEM in splenocyte proliferative responses and splenocyte activation with ex vivo restimulated splenocytes, we next detected whether RLP_3_-GEM with GEL01 could also promote cytokine secretion profiles in immunized mice. Splenocytes were collected from immunized mice and were ex vivo restimulated with RBD; then, the Th1 (IFN-r, TNF-a, and IL-2) and Th2 (IL-4, IL-6, and IL-10) cytokines in the supernatants were detected by ELISA. The levels of these cytokines in the RLP_3_-GEM with GEL01 group were significantly higher than those in the RLP_3_-GEM group (Figure 6). The ELISpot assay showed that the amount of splenocytes secreting IFN-r and IL-4 was also remarkably increased in the RLP_3_-GEM with GEL01 group, similar to the results of the ELISA. Overall, the above data showed that RLP_3_-GEM with GEL01 induced the secretion of the Th1 and Th2 cytokines, suggesting a stronger immune response than RLP_3_-GEM.

## 4. Discussion

Because MERS-CoV causes human respiratory infections with a high mortality rate and human-to-human transmission [41], the development of effective MERS-CoV vaccines, especially subunit-based vaccines, will provide the safest way of preventing the continuous dissemination of MERS-CoV in humans and camels [21]. Previous results showed that MERS-CoV RBD-based subunit vaccines have displayed effective immunogenicity against MERS-CoV challenges in mice [6,22,24]. Some studies have shown that the recombinant RBD of MERS-CoV spike protein with intranasal vaccination induced more robust local mucosal immune responses in the lung and stronger systemic cellular immune responses than that with subcutaneous vaccination in mice, demonstrating that intranasal RBD-based subunit vaccines are a safe and effective means to prevent MERS-CoV infections [42]. A study demonstrated that MERS-CoV could replicate robustly in human primary intestinal epithelial cells, intestinal organoids, and small intestine explants and could resist fed-state gastrointestinal fluids, causing enteric MERS-CoV infection [8]. Therefore, it is extremely important for MERS-CoV vaccination to induce respiratory and gastrointestinal mucosal immunity. In this study, we developed RBD-based MERS-CoV subunit vaccines with a novel bacterial antigen display system and evaluated the immunogenicity of specific mucosal immunity in lung and intestinal mucosa and the systemic immune responses due to immunization via the intranasal route.

A novel method for surface display of an antigen is based on the nonrecombinant and nonliving *Lactococcus lactis* bacteria-assigned GEM particles. *Lactococcus lactis* has a long history of use in foods and is recognized as safe [43]. The particles boiled in acids mainly consist of bacterial-shaped peptidoglycan spheres, lack other cell wall components and intracellular materials and are recognized as being especially safe [28]. The particles can bind to exogenous antigens by a PA with a high affinity and a high loading capacity [30]. In addition to the above advantages, the particles can bind to the chimeric anchor fusion proteins in culture medium at room temperature in an efficient, strong, and selective manner; the particles can only need a one-step centrifugation process to obtain the purified fusion proteins without the extra purification steps [31]; it is easier to obtain purified antigens in a bacterium-like particles vaccine compared with the virus-like particles vaccine. The GEM-PA system has been applied in a variety of animal models and has shown strong antigen-specific systemic immune responses with parenteral vaccination and both robust local and systemic responses induced by mucosal vaccination; this system has even proven to be protective against specific pathogens, including parasites, bacteria and viruses [33,44,45,46,47,48]. Our results showed that the MERS-CoV RBD proteins were successfully displayed on the surface of the GEM particles with the GEM-PA system.

In this GEM-PA system, PA is the LysM from the C-terminal peptidoglycan-binding domain of AcmA, an autolysin from *Lactococcus lactis*, and binds to GEM particles in a noncovalent manner. One of the important factors determining the binding activity of fusion proteins is the number of LysMs. In the wild, the number of LysM domains in the proteins differs greatly [49]. In the current study, the optimal number of LysM domains in the GEM-PA system for proper function are different in different groups [30,36,37]. The possible reason for this was that fusion proteins fused to different target proteins. Our results showed that the binding activity of RLP_3_ was remarkably higher than that of RLP_2_ in GEM particles, which is consistent with the results of Kenji Okano and Anton Steen [36,50]. LysM domains are often separated from the other domains and from each other through linker sequences, raising speculation about LysM domain mobility and flexibility to the fused target proteins and allowing the proteins to attain an optimal orientation in binding with the cell wall [35]. Our results showed that the binding activity of RLP_3_ was slightly higher than that of RP_3_. Furthermore, there were significant differences in the neutralizing activity among the sera of mice intramuscularly immunized with RLP_3_-GEM and RP_3_-GEM. These results clearly suggested that the linker peptides between the PA and RBD proteins could increase the binding capacity of the PA and the immunogenicity of the RBD in the fusion proteins. Consistent with those results, linkers in the recombinant fusion proteins could improve the biological activity of functional domains [51]. Thus, this study represents a significant advancement in the development of the GEM-PA system.

MERS-CoV transmission from camels to humans is via respiratory droplets or saliva during direct contact with infected camels or through the consumption of contaminated camel milk or meat [8]. A more effective MERS-CoV vaccine should not only provide protection against the invasion of MERS-CoV in the blood but also provide protection against invasive infections in the respiratory tract and gastrointestinal tract. Therefore, developing an MERS-CoV alternative vaccine with mucosal immune responses is necessary. In this study, we further demonstrated that RLP_3_-GEM was a promising candidate for intranasal immunization with the MERS-CoV subunit vaccine. Moreover, we used GEL01 as an adjuvant for intranasal vaccination. This is because GEL01, a polymer of a sodium polyacrylate-based adjuvant, can enhance the protective effects of live vaccines through mucosal immunity [52]. Our results showed that RLP_3_-GEM (PLPs) induced strong neutralization against MERS-CoV pseudotyped virus infections and RBD-specific IgG, IgG1, and IgG2a antibody responses in sera, and these responses also significantly increased in the presence of the GEL01 adjuvant. IgG2a is the most effective in activating immune cells, and the ratios of IgG2a/IgG1 indicate Th1/Th2 polarization [53]. Moreover, the ratio of IgG2a/IgG1 for IgG induced by BLPs plus GEL01 was higher than that of the BLPs alone, suggesting that GEL01 showed adjuvanticity to induce a Th1-polarized immune response. SIgA antibodies may limit MERS-CoV replication at mucosal surfaces. Our data showed that intranasally immunized BLPs could induce mucosal sIgA, especially in the small intestine, confirming that the BLP vaccine could induce strong local mucosal immunity via the intranasal route. To our knowledge, this is the first report that showed successful generation of sIgA in the small intestine among the published MERS-CoV vaccine candidates.

Furthermore, we evaluated splenocytes by ex vivo restimulation, detecting splenocyte proliferation, splenocyte activation and cytokine secretion. The results showed that the BLP vaccine could increase splenocyte proliferation. CD69 is an early marker of activated immune cells [54]. We observed that B cells, CD4^+^ T cells and CD8^+^ T cells from immunized mouse splenocytes activated more rapidly than those from mock mice once restimulated by antigen. Next, Th1 (IFN-γ, TNF-α and IL-2) and Th2 (IL-4, IL-6 and IL-10) in the splenocyte supernatants were detected by ELISA and/or ELISpot. The levels from the BLP vaccine in the presence of the GEL01 adjuvant were higher than those from the BLP vaccine alone. Taken together, GEL01 as a BLP adjuvant can strongly induce systemic and local mucosal immunity. This may be because sodium polyacrylate, the mainly component of the GEL01 adjuvant, promotes the retention of antigens and sustains the release of antigens in the nasal mucosa; in addition, this adjuvant recruits immune cells to the nasal mucosa by the inflammatory response [55].

In summary, we constructed a MERS-CoV bacterium-like particle vaccine displaying the RBD antigen protein in this study. Our results clearly demonstrated that the vaccine can induce strong, specific mucosal immunity in the lungs and intestinal tract and systemic immune responses with the GEL01 adjuvant via the intranasal route. GEL01-adjuvanted MERS-CoV bacterium-like particles are a promising candidate vaccine, and a protective efficacy evaluation of the vaccine in animal models will be considered for future studies.

## Figures and Tables

**Table 1:** Oligonucleotide primers used in this study.

Oligonucleotide Primers	Sequences
RBD-F^1,3^	5′-TGC*TCTAGA***CATCACCATCACCATCAC**CAAGCCGAAGGAGTTGAA-3′ (XbaI)
Linker-RBD-R^2^	5′-ACCAGAACCACCACCAGAACCACCCAACTTAGGGCAGACGCT-3′
RBD-R	5′-GTTACCAGCTGAAGAAGCACCATCCAACTTAGGGCAGACGCT-3′
PA3-F	5′-ACCAATAGCGTCTGCCCTAAGTTGGATGGTGCTTCTTCAGCTGG-3′
Linker-PA2-F^2^	5′-GGTGGTTCTGGTGGTGGTTCTGGTACTACCGTTAAGGTGAAGTC-3′
Linker-PA3-F^2^	5′-GGTGGTTCTGGTGGTGGTTCTGGTGATGGTGCTTCTTCAGCTGG-3′
PA3-R^1^	5′-CGG*GGTACC*TTACTTGATACGCAGGTATTGAC-3′ (KpnI)

^1^ restriction enzyme sites are underlined and italicized. ^2^ Middle linker (Gly-Gly-Ser-Gly)x2 base sequences are underlined. ^3^ His-tag base sequences are in bold.
